# The Multivariate Temporal Response Function (mTRF) Toolbox: A MATLAB Toolbox for Relating Neural Signals to Continuous Stimuli

**DOI:** 10.3389/fnhum.2016.00604

**Published:** 2016-11-30

**Authors:** Michael J. Crosse, Giovanni M. Di Liberto, Adam Bednar, Edmund C. Lalor

**Affiliations:** ^1^School of Engineering, Trinity Centre for Bioengineering and Trinity College Institute of Neuroscience, Trinity College DublinDublin, Ireland; ^2^Department of Pediatrics and Department of Neuroscience, Albert Einstein College of MedicineThe Bronx, NY, USA; ^3^Department of Biomedical Engineering and Department of Neuroscience, University of RochesterRochester, NY, USA

**Keywords:** system identification, reverse correlation, stimulus reconstruction, sensory processing, EEG/MEG

## Abstract

Understanding how brains process sensory signals in natural environments is one of the key goals of twenty-first century neuroscience. While brain imaging and invasive electrophysiology will play key roles in this endeavor, there is also an important role to be played by noninvasive, macroscopic techniques with high temporal resolution such as electro- and magnetoencephalography. But challenges exist in determining how best to analyze such complex, time-varying neural responses to complex, time-varying and multivariate natural sensory stimuli. There has been a long history of applying system identification techniques to relate the firing activity of neurons to complex sensory stimuli and such techniques are now seeing increased application to EEG and MEG data. One particular example involves fitting a filter—often referred to as a temporal response function—that describes a mapping between some feature(s) of a sensory stimulus and the neural response. Here, we first briefly review the history of these system identification approaches and describe a specific technique for deriving temporal response functions known as regularized linear regression. We then introduce a new open-source toolbox for performing this analysis. We describe how it can be used to derive (multivariate) temporal response functions describing a mapping between stimulus and response in both directions. We also explain the importance of regularizing the analysis and how this regularization can be optimized for a particular dataset. We then outline specifically how the toolbox implements these analyses and provide several examples of the types of results that the toolbox can produce. Finally, we consider some of the limitations of the toolbox and opportunities for future development and application.

## Introduction

Traditionally, research on the electrophysiology of sensory processing in humans has focused on the rather special case of brief, isolated stimuli because of the need to time-lock to discrete sensory events in order to estimate event-related potentials (ERPs; Luck, Handy, 2005; 2014). The objective is to estimate the impulse response function of the sensory system under investigation by convolving the system with a transient, impulse-like stimulus and averaging over several-hundred time-locked response trials. This approach has been used extensively to study how the human brain processes various ecological events, even those that occur in a continuous, dynamic manner such as human speech (e.g., Salmelin, 2007; Picton, 2013). However, the type of speech stimuli used in such ERP studies usually consist of individual phonemes or syllables and are therefore not entirely reflective of natural, connected speech which is ongoing and abundant with lexical complexity. Recent studies have begun to use more naturalistic, extended speech stimuli by focusing their analysis on measuring the phase of neural responses across multiple repetitions of the same speech segment (Luo and Poeppel, 2007; Zion-Golumbic et al., 2013). While this approach has revealed novel and important insights into the neurophysiology of speech processing, it does not facilitate characterization of the system's response function, and in any case, is an indirect measure of how the brain entrains to the stimulus over time.

A more direct way to investigate neural entrainment to continuous stimuli is to mathematically model a function that describes the way a particular property of the stimulus is mapped onto neural responses, a technique known as system identification (SI; Marmarelis, 2004). While there are several classes of models that can be implemented for this purpose (reviewed in Wu et al., 2006), the most straightforward class are linear time-invariant (LTI) systems. Although the human brain is neither linear nor time-invariant, these assumptions can be reasonable in certain cases (e.g., Boynton et al., 1996) and allow for the system to be characterized by its impulse response. An SI method known as “reverse correlation” has become a common technique for characterizing LTI systems in neurophysiology (Ringach and Shapley, 2004), an approach that has long been established in both visual and auditory animal electrophysiology (De Boer and Kuyper, 1968; Marmarelis and Marmarelis, 1978; Coppola, 1979). This technique approximates the impulse response of the sensory system under investigation, except it does not require the use of discrete stimuli. While this is somewhat analogous to calculating an ERP, there are important differences that must be considered: (1) the response function obtained by reverse correlation only reflects the response of the system to specific stimulus parameters defined by the experimenter as opposed to the entire event, (2) reverse correlation makes the assumption that the input-output relationship of the system is linear, unlike time-locked averaging and (3) reverse correlation converges on a more temporally precise estimate of the systems impulse response than an ERP (which is susceptible to temporal smearing). Reverse correlation in its simplest form can be implemented via a straightforward cross-correlation between the input and output of an LTI system (Ringach and Shapley, 2004). While this approach has been used to study how speech is encoded in human brain activity (Ahissar et al., 2001; Abrams et al., 2008; Aiken and Picton, 2008), it is better suited to stimuli modulated by a stochastic process such as Gaussian white noise. As such, most instances of this approach in animal models have traditionally used white noise stimuli (De Boer and Kuyper, 1968; Marmarelis and Marmarelis, 1978; Coppola, 1979; Eggermont et al., 1983; Ringach et al., 1997). This work has even inspired researchers to investigate how such stochastic signals are encoded in the human brain (Lalor et al., 2006, 2009).

That said, the human brain has evolved to process ecologically relevant stimuli that rarely conform to a white random process. For example, in the context of human neuroscience research, a proper understanding of how the brain processes natural speech would surely require that natural speech is used as a stimulus in the laboratory, given that neurons respond differently to more complex stimuli (Theunissen et al., 2000). As such, researchers using animal models have shifted their focus toward studying the brain using more naturalistic stimuli thanks to the development of SI methods such as “normalized reverse correlation” (NRC; Theunissen et al., 2001), “ridge regression” (Machens et al., 2004), and “boosting” (David et al., 2007). Each of these techniques converge on the same theoretical solution but use different priors and, critically, give an unbiased impulse response estimate for non-white stimuli. This has inspired researchers to characterize the “spectrotemporal receptive fields” of auditory cortical neurons in various animal models (Depireux et al., 2001; Tomita and Eggermont, 2005). As a result, researchers interested in how human speech is processed have begun to model response functions describing the linear mapping between properties of natural speech (such as the envelope or spectrogram) and population responses in both animals (David et al., 2007; Mesgarani et al., 2008) and humans (Lalor and Foxe, 2010; Ding and Simon, 2012b). There have been similar efforts to model response functions relating more natural visual stimulus properties such as motion to neural responses in humans (Gonçalves et al., 2014), again inspired by previous single-unit electrophysiology work (Jones and Palmer, 1987; David and Gallant, 2005).

Most of the aforementioned studies have modeled the stimulus-response mapping function in the forward direction (i.e., forward modeling). However, this mapping can also be modeled in the reverse direction (i.e., backward modeling), offering a complementary way to investigate how stimulus features are encoded in neural response measures. Unlike forward models, backward model parameters are not readily neurophysiologically interpretable (see Haufe et al., 2014), but can be used to reconstruct or decode stimulus features from the neural response, a method known as “stimulus reconstruction.” This approach has several advantages over forward modeling approaches, especially when recording from population responses using multi-channel systems such as EEG. Firstly, because reconstruction projects back to the stimulus domain, it does not require pre-selection of neural response channels (Mesgarani et al., 2009). In fact, inclusion of all response channels in the backward model is advantageous because the reconstruction method gives a low weighting to irrelevant channels whilst allowing the model to capture additional variance using channels potentially excluded by feature selection approaches (Pasley et al., 2012). Secondly, backward modeling can offer increased sensitivity to important signal differences between response channels that are highly correlated with each other (as is often the case with EEG). It can do this because the analysis maps the data from all response channels simultaneously (i.e., in a multivariate manner) and so it can recognize any inter-channel correlation in the data (Mesgarani et al., 2009). In contrast, when performing forward modeling, each analysis is univariate and thus is ignorant of the data on the other EEG channels. Thirdly, stimulus features that are not explicitly encoded in the neural response may be inferred from correlated input features that are encoded. This prevents the model from allocating resources to the encoding of redundant stimulus information (Barlow, 1972). The stimulus reconstruction method has previously been used to study both the visual and auditory system in various animal models (Bialek et al., 1991; Rieke et al., 1995; Stanley et al., 1999). More recently, it has been adopted for studying speech processing in the human brain using intracranial and non-invasive electrophysiology (Mesgarani et al., 2009; Pasley et al., 2012; Ding and Simon, 2013; Martin et al., 2014; Crosse et al., 2015a, Crosse et al., 2016; O'Sullivan et al., 2015).

While certain research groups now regularly use SI to study sensory processing in the human brain, the approach has perhaps not yet been as widely adopted throughout the neuroscience community as it might because of the (at least perceived) challenges associated with its implementation. The goal of the present paper is to introduce a recently-developed SI toolbox that provides a straightforward and flexible implementation of regularized linear (ridge) regression (Machens et al., 2004; Lalor et al., 2006). We begin by summarizing the mathematics underlying this technique, continue by providing some concrete examples of how the toolbox can be used and conclude by discussing some of its applications and important considerations.

## Regularized linear regression

### Forward models: temporal response function estimation

Forward models are sometimes referred to as generative or encoding models because they describe how the system generates or encodes information (Haufe et al., 2014). Here, they will be referred to as temporal response functions (TRFs; Ding and Simon, 2012b). There are a number of ways of mathematically describing how the input to a system relates to its output. One commonly used approach—and the one that will be described in this paper—is to assume that the output of the system is related to the input via a simple linear convolution. In the context of a sensory system where the output is monitored by *N* recording channels, let's assume that the instantaneous neural response *r*(*t, n*), sampled at times *t* = 1…*T* and at channel *n*, consists of a convolution of the stimulus property, *s*(*t*), with an unknown channel-specific TRF, *w*(τ, *n*). The response model can be represented in discrete time as:

(1) $$r(t,n)=\sum_{\tau }w(\tau ,n)s(t-\tau )+\varepsilon (t,n),$$

where ε(*t, n*) is the residual response at each channel not explained by the model. Essentially, a TRF can be thought of as a filter that describes the linear transformation of the ongoing stimulus to the ongoing neural response. The TRF, *w*(τ, *n*), describes this transformation for a specified range of time lags, τ, relative to the instantaneous occurrence of the stimulus feature, *s*(*t*).

In the context of speech for example, *s*(*t*) could be a measure of the speech envelope at each moment in time and *r*(*t, n*) could be the corresponding EEG response at channel *n*. The range of time lags over which to calculate *w*(τ, *n*) might be that typically used to capture the cortical response components of an ERP, e.g., −100–400 ms. The resulting value of the TRF at −100 ms, would index the relationship between the speech envelope and the neural response 100 ms earlier (obviously this should have an amplitude of zero), whereas the TRF at 100 ms would index how a unit change in the amplitude of the speech envelope would affect the EEG 100 ms later (Lalor et al., 2009).

The TRF, *w*(τ, *n*), is estimated by minimizing the mean-squared error (MSE) between the actual neural response, *r*(*t, n*), and that predicted by the convolution, $\hat{r}\left(t,n\right)$:

(2) $$\min\varepsilon (t,n)=\sum_{t}{\left[r(t,n)-\hat{r}(t,n)\right]}^{2}.$$

In practice, this is solved using reverse correlation (De Boer and Kuyper, 1968), which can be easily implemented using the following matrix operations:

(3) $$w={\left(S^{\text{T}}S\right)}^{-1}S^{\text{T}}r,$$

where **S** is the lagged time series of the stimulus property, s, and is defined as follows:

(4) $$S=\left[\begin{array}{ccccccc} s(1-\tau_{\min}) & s(-\tau_{\min}) & \cdots & s(1) & 0 & \cdots & 0\\ \vdots & \vdots & \cdots & \vdots & s(1) & \cdots & \vdots \\ \vdots & \vdots & \cdots & \vdots & \vdots & \cdots & 0\\ \vdots & \vdots & \cdots & \vdots & \vdots & \cdots & s(1)\\ s(T) & \vdots & \cdots & \vdots & \vdots & \cdots & \vdots \\ 0 & s(T) & \cdots & \vdots & \vdots & \cdots & \vdots \\ \vdots & 0 & \cdots & \vdots & \vdots & \cdots & \vdots \\ \vdots & \vdots & \cdots & \vdots & \vdots & \cdots & \vdots \\ 0 & 0 & \cdots & s(T) & s(T-1) & \cdots & s(T-\tau_{\max}) \end{array}\right].$$

The values τ_min_ and τ_max_ represent the minimum and maximum time lags (in samples) respectively. In **S**, each time lag is arranged column-wise and non-zero lags are padded with zeros to ensure causality (Mesgarani et al., 2009). The window over which the TRF is calculated is defined as τ_*window*_ = τ_max_ − τ_min_ and the dimensions of **S** are thus *T* × τ_*window*_. To include the constant term (y-intercept) in the regression model, a column of ones is concatenated to the left of **S**. In Equation (3), variable **r** is a matrix containing all the neural response data, with channels arranged column-wise (i.e., a *T* × *N* matrix). The resulting TRF, **w**, is a τ_*window*_ × *N* matrix with each column representing the univariate mapping from **s** to the neural response at each channel.

One of the important points here is that this analysis explicitly takes into account the autocovariance structure of the stimulus. In non-white stimuli, such as natural speech, the intensity of the acoustic signal modulates gradually over time, meaning it is correlated with itself at non-zero time lags. A simple cross-correlation of a speech envelope and the corresponding neural response would result in temporal smearing of the impulse response function. The solution here (Equation 3) is to divide out the autocovariance structure of the stimulus from the model such that it removes the correlation between different time points. The TRF approach, which does this, is therefore less prone to temporal smearing than a simple cross-correlation approach. This is demonstrated in a worked example in the next section.

### Regularization

An important consideration when calculating the TRF is that of regularization, i.e., introducing additional information to solve any ill-posed estimation problems and prevent overfitting. The ill-posed estimation problem has to do with inverting the autocovariance matrix, **S**^T^**S**. Matrix inversion is particularly prone to numerical instability when solved with finite precision. In other words, small changes in **S**^T^**S** (such as rounding errors due to discretization) could cause large changes in **w** if the former is ill-conditioned. In other words, the estimate of **w** can have very high variance. This does not usually apply when the stimulus represents a stochastic process because **S**^T^**S** would be full rank (Lalor et al., 2006). However, the autocorrelation properties of a non-white stimulus such as speech means that it is more likely to be singular (i.e., have a determinant of zero). Typically, numerical treatment of an ill-conditioned matrix involves reducing the variance of the estimate by adding a bias term or “smoothing solution.” Specifically, because the overall estimation error is made up of both a bias term (i.e., the difference between the estimate's expected value and its true value) and a variance term, one can deliberately increase the bias so as to reduce the (high) variance of the estimate by so much as to decrease the overall estimation error.

Addition of this smoothing term also solves the other main issue, that of overfitting. The reverse correlation analysis is utterly agnostic as to the biological nature of the data that it is being asked to model. As a result, without regularization, the resulting TRF will be optimal in terms of the particular fitting criterion (e.g., least squares error) for the specific dataset that was used for the fitting. And, given that those data will be “noisy,” the TRF can display biologically implausible properties such as very high-frequency fluctuations. Using this TRF to then predict unseen data will likely result in suboptimal performance, because the high frequency fluctuations will not necessarily correspond well to the “noise” in the new data. In other words, the TRF has been “overfit” to the specific dataset used in the training. Regularization serves to prevent overfitting to such high-frequency, dataset-specific noise along the low-variance dimensions (Theunissen et al., 2001; Mesgarani et al., 2008). It can do this, for example, by penalizing large differences between neighboring TRF values, thereby forcing the TRF to be smoother. This makes the TRF less specific to the data that was used to fit it and can help it generalize better to new unseen data.

In practice, both ill-posed problems and overfitting can be solved simultaneously by weighting the diagonal of **S**^T^**S** before inversion, a method known as Tikhonov regularization or ridge regression (Tikhonov and Arsenin, 1977):

(5) $$w={\left(S^{\text{T}}S+\lambda I\right)}^{-1}S^{\text{T}}r,$$

where **I** is the identity matrix and λ is the smoothing constant or “ridge parameter.” The ridge parameter can be adjusted using cross-validation to maximize the correlation between *r*(*t, n*), and $\hat{r}\left(t,n\right)$ (David and Gallant, 2005). TRF optimization will be described in more detail in the next section. While this form of ridge regression enforces a smoothness constraint on the resulting model by penalizing TRF values as a function of their distance from zero, another option is to quadratically penalize the difference between each two neighboring terms of **w** (Lalor et al., 2006):

(6) $$w={\left(S^{T}S+\lambda M\right)}^{-1}S^{T}r,\text{where }M=\left[\begin{array}{cccccc} 1 & -1 & & & & \\ -1 & 2 & -1 & & & \\ & -1 & 2 & -1 & & \\ & & \ddots & \ddots & \ddots & \\ & & & -1 & 2 & -1\\ & & & & -1 & 1 \end{array}\right].$$

Tikhonov regularization (Equation 5) reduces overfitting by smoothing the TRF estimate in a way that is insensitive to the amplitude of the signal of interest. However, the quadratic approach (Equation 6) reduces off-sample error whilst preserving signal amplitude (Lalor et al., 2006). As a result, this approach usually leads to an improved estimate of the system's response (as indexed by MSE) compared to Tikhonov regularization.

### Multivariate analysis

The previous section focused on the specific case of relating a single, univariate input stimulus feature (e.g., the envelope of a speech stimulus) separately to each of multiple recording channels. However, most complex stimuli in nature are not processed as simple univariate features. For example, when auditory speech enters the ear, the signal is transformed into a spectrogram representation by the cochlea, consisting of multiple frequency bands which project along the auditory pathway (Yang et al., 1992). The auditory system maps each of these frequency bands to the neural representation measured at the cortical level. This process can be modeled by a multivariate form of the TRF (i.e., mTRF).

Indeed, it is possible to define an mTRF that linearly maps a multivariate stimulus feature to each recording channel (Theunissen et al., 2000; Depireux et al., 2001). Using the above example, let *s*(*t, f*) represent the spectrogram of a speech signal at frequency band *f* = 1…*F*. To derive the mTRF, the stimulus lag matrix, **S** (Equation 4), is simply extended such that every column is replaced with *F* columns, each representing a different frequency band (i.e., a *T* × *Fτ*_*window*_ matrix). The resulting mTRF, *w*(*f*, τ, *n*), will be a *Fτ*_*window*_ × *N* matrix but can easily be unwrapped such that each independent variable is represented as a separate dimension (i.e., a *F* × τ_*window*_ × *N* matrix). Here, the constant term is included by concatenating *F* columns to the left of **S**.

An important consideration in multivariate TRF analysis is which method of regularization to use. The quadratic regularization term in Equation (6) was designed to enforce a smoothness constraint and maintain SNR along the time dimension, but not any other. For high λ values, this approach would cause smearing across frequencies; hence it would not yield an accurate representation of the TRF in each frequency band. In this case, it will typically be most appropriate to use the identity matrix for regularization (Equation 5) so as to avoid enforcing a smoothness constraint across the non-time dimension of the mTRF—although, in some cases, this may actually be what is desired.

### Backward models: stimulus reconstruction

The previous sections describe how to forward model the linear mapping between the stimulus and the neural response. While this approach can be extended to accommodate multivariate stimulus features, it is suboptimal in the sense that it treats each neural response channel as an independent univariate feature. Backward modeling, on the other hand, derives a reverse stimulus-response mapping by exploiting all of the available neural data in a multivariate context. Backward models are sometimes referred to as discriminative or decoding models, because they attempt to reverse the data generating process by decoding the stimulus features from the neural response (Haufe et al., 2014). Here, they will simply be referred to as decoders.

Decoders can be modeled in much the same way as TRFs. Suppose the decoder, *g*(τ, *n*), represents the linear mapping from the neural response, *r*(*t, n*), back to the stimulus, *s*(*t*). This could be expressed in discrete time as:

(7) $$\hat{s}(t)=\sum_{n}\sum_{\tau }r(t+\tau ,n)g(\tau ,n),$$

where ŝ(*t*) is the reconstructed stimulus property. Here, the decoder integrates the neural response over a specified range of time lags τ. Ideally, these lags will capture the window of neural data that optimizes reconstruction of the stimulus property. Typically, the most informative lags for reconstruction are commensurate with those used to capture the major components of a forward TRF, except in the reverse direction as the decoder effectively maps backwards in time. To reverse the lags used in the earlier TRF example (τ_min_ = −100 ms, τ_max_ = 400 ms), the values of τ_min_ and τ_max_ are swapped but their signs remain unchanged, i.e., τ_min_ = −400 ms, τ_max_ = 100 ms.

The decoder, *g*(τ, *n*), is estimated by minimizing the MSE between *s*(*t*) and ŝ(*t*):

(8) $$\min\varepsilon (t)=\sum_{t}{\left[s(t)-\hat{s}(t)\right]}^{2}.$$

Analogous to the TRF approach, the decoder is computed using the following matrix operations:

(9) $$g={\left(R^{\text{T}}R+\lambda I\right)}^{-1}R^{\text{T}}s$$

where **R** is the lagged time series of the response matrix, **r**. For simplicity, we will define **R** for a single-channel response system:

(10) $$R=\left[\begin{array}{ccccccc} r(1-\tau_{\min},\text{​}1) & r(-\tau_{\min},\text{​}1) & \cdots & r(1,\text{​}1) & 0 & \cdots & 0\\ \vdots & \vdots & \cdots & \vdots & r(1,1) & \cdots & \vdots \\ \vdots & \vdots & \cdots & \vdots & \vdots & \cdots & 0\\ \vdots & \vdots & \cdots & \vdots & \vdots & \cdots & r(1,\text{​}1)\\ r(T,\text{​}1) & \vdots & \cdots & \vdots & \vdots & \cdots & \vdots \\ 0 & r(T,\text{​}1) & \cdots & \vdots & \vdots & \cdots & \vdots \\ \vdots & 0 & \cdots & \vdots & \vdots & \cdots & \vdots \\ \vdots & \vdots & \cdots & \vdots & \vdots & \cdots & \vdots \\ 0 & 0 & \cdots & r(T,\text{​}1) & r(T-1,\text{​}1) & \cdots & r(T-\tau_{\max},\text{​}1) \end{array}\right],$$

As before, this can be extended to the multivariate case of an *N*-channel system by replacing each column of **R** with *N* columns (each representing a separate recording channel). For *N* channels, the dimensions of **R** would be *T* × *Nτ*_*window*_. The constant term is included by concatenating *N* columns of ones to the left of **R**. In the context of speech, the stimulus variable, **s**, represents either a column-wise vector (e.g., envelope) or a *T* × *F* matrix (e.g., spectrogram). The resulting decoder, **g**, would be a vector of *Nτ*_*window*_ samples or a *Nτ*_*window*_ × *F* matrix, respectively. While interpretation of decoder weights is not as straightforward as that of a TRF, one may wish to separate its dimensions (e.g., *N* × τ_*window*_ × *F*) to examine the relative weighting of each channel at a specific time lag. The channel weights represent the amount of information that each channel provides for reconstruction, i.e., highly informative channels receive weights of greater magnitude while channels providing little or no information receive weights closer to zero.

In Equation (9), Tikhonov regularization is used as it is assumed that the neural response data is multivariate. As mentioned above, any bias from the correlation between the neural response channels is removed in the reconstruction approach. In practice, this is achieved by dividing out the autocovariance structure of the neural response (see Equation 9). As a result, channel weighting becomes much more localized because inter-channel redundancies are no longer encoded in the model, giving it an advantage over the forward TRF method and cross-correlation approaches.

## mTRF toolbox: implementation and functionality

This section outlines how regularized linear regression can be implemented in MATLAB using the mTRF Toolbox (https://sourceforge.net/projects/aespa/). Specifically, it describes how to train and test on univariate and multivariate datasets and how the resulting model should be optimized for specific purposes.

### Training

Modeling the stimulus-response mapping of a given dataset is implemented in the mTRF Toolbox using a simple function called *mTRFtrain*. This function computes univariate or multivariate ridge regression as described in the previous section (Equations 5, 6, and 9). The model can be trained on the data set in two separate ways: (1) by training on each trial separately and averaging over *M* models, or (2) by training on a concatenation of trials. Both of these approaches yield the same results because the data are modeled using a linear assumption. Here, the former approach will be considered because it affords certain advantages. Firstly, by generating separate models for each of the *M* trials, certain denoising algorithms that require repetition of “trials” can be applied to the model coefficients, even if they were modeled on different stimuli, e.g., joint decorrelation (de Cheveigné and Parra, 2014). Secondly, artifacts from discontinuities between trials are not an issue. Thirdly, cross-validation is much more efficient because training models on small amounts of data and averaging across trials is much faster than concatenating large amounts of data and training on them.

**Figure 1:**
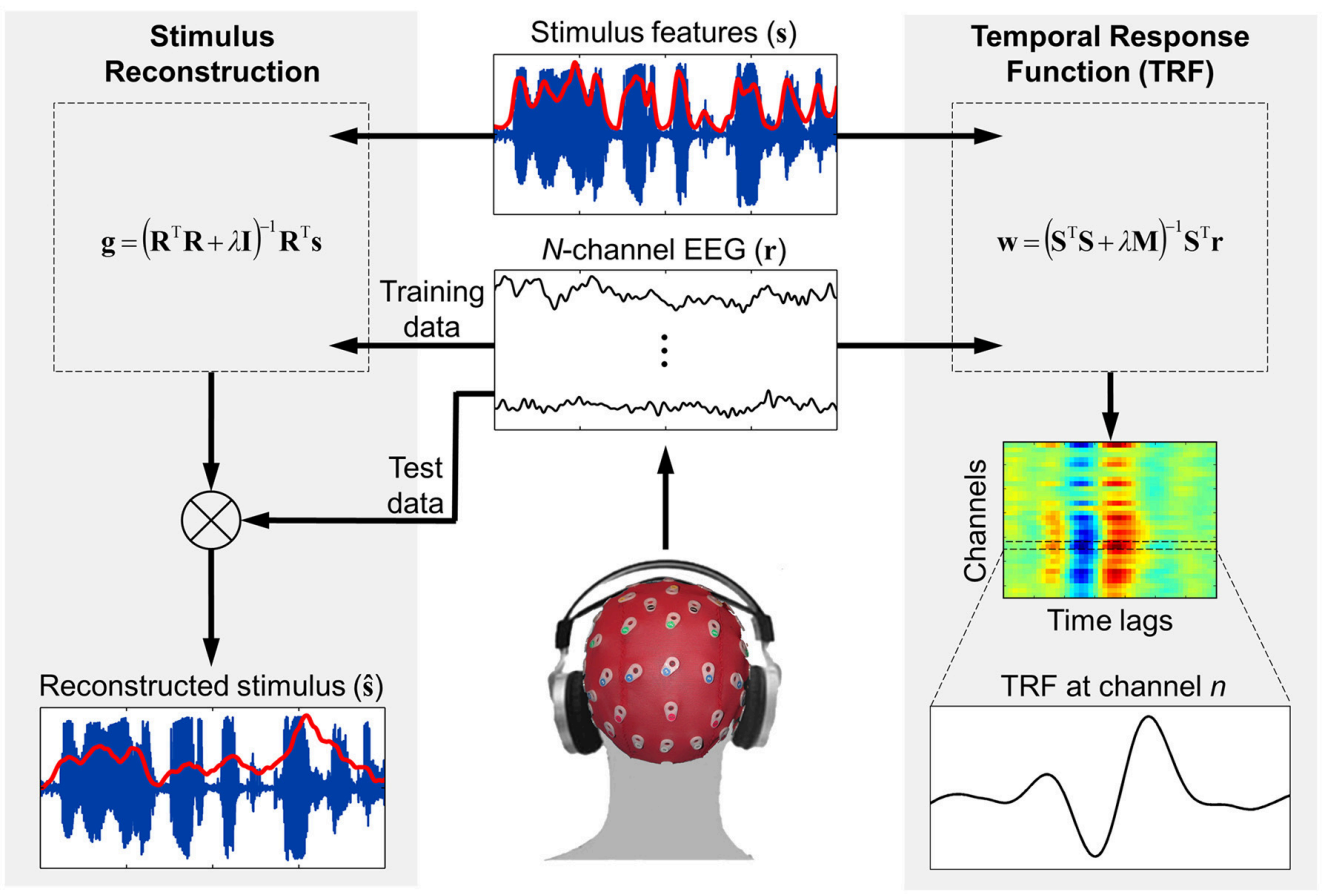
**Schematic of the forward and backward modeling approaches implemented by mTRF Toolbox**. Stimulus reconstruction (i.e., backward modeling) can be used to decode specific stimulus features from recorded neural response data in order to estimate how accurately this information was encoded in the brain. Temporal response function estimation (i.e., forward modeling) can be used in a similar manner to predict the neural response to a novel stimulus, but also allows for detailed examination of how the stimulus features were encoded in the brain and interpretation of the underlying neural generators.

For a given trial, the *mTRFtrain* function trains on all data features (e.g., frequency bands, response channels) simultaneously (see Figure 1). The only requirement is that the stimulus and response data have the same sampling rate (which is specified in Hz) and be the same length in time. As described in the previous section, vectors and matrices should be organized such that all features are arranged column-wise. The mapping direction is specified as “1” (forward modeling) or “−1” (backward modeling). The minimum and maximum time lags are entered in milliseconds and converted to samples based on the sampling rate entered. It is often useful to include additional time lags such as prestimulus lags for visualization purposes. And one should also be aware of regression artifacts at either extreme of the resulting model. However, when optimizing models for prediction purposes, it is advisable to use only stimulus-relevant time lags. The lag matrix used in the ridge regression is generated by a function called *lagGen*. If the user specifies to map backwards, the lags are automatically reversed and the algorithm is changed from Equations (5) to (9). If the stimulus entered is univariate (i.e., a vector), the algorithm will automatically switch to Equation 6 to use the superior quadratic ridge penalty. The final parameter that must be specified is the ridge parameter, λ. For visualization of model coefficients, λ can be empirically chosen as the lowest value such that any increase would result in no visible improvement in the plotted estimate (Lalor et al., 2006). For optimizing model performance, a more systematic approach should be implemented such as cross-validation, as described in the following section.

### Optimization

Optimization of the stimulus-response mapping can be achieved via cross-validation and is implemented using the *mTRFcrossval* function. Specifically, the goal is to identify the value of the ridge parameter that optimizes this mapping. Here, the entire dataset is entered together, with *M* stimuli and *M* response matrices arranged in two cell arrays. There is no requirement that the individual trials be the same length in time (although this is preferable for optimization reasons). Another important factor that optimizes cross-validation is normalization of both input and output data. By z-scoring the data, the range of values needed to conduct a comprehensive parameter search can be greatly reduced, making the process more efficient. The ridge values over which validation is measured can be entered as a single vector. All other parameters are entered in the same way as in *mTRFtrain*.

The validation approach implemented in *mTRFcrossval* is that of “leave-one-out” cross-validation, although this could also be described as *M*-fold cross-validation. First, a separate model is fit to each of the *M* trials for every ridge value specified. Then, the trials are rotated *M* times such that each trial is “left out” or used as the “test set,” and the remaining *M*−1 trials are assigned as the “training set” (see Figure 1). The actual models tested are obtained by averaging over the single-trial models assigned to each training set. As mentioned earlier, this approach is more efficient than concatenating *M*−1 trials and fitting a model to these data. Each averaged model is then convolved with data from the corresponding test set to predict either the neural response (forward modeling) or the stimulus signal (backward modeling). This process is repeated for each of the different ridge values. Validation of the model is assessed by comparing the predicted estimate with the corresponding original data. Two different validation metrics are used: Pearson's correlation coefficient and mean squared error. Once the validation metrics have been obtained, they should be averaged across all trials. This approach is advisable because each of the models should in theory require the same ridge value for regularization, given that they share *M*−2 trials of data with each other. This ensures that the models generalize well to new data and are not overfit to the test set. However, this approach works best if all the trials are the same length. The optimal ridge value is identified as that which yields either the highest *r*-value or the lowest MSE-score on average.

### Testing

Once the model parameters have been tuned using cross-validation, the optimized model can be tested on new data using the *mTRFpredict* function. This can be conducted on data that was held aside from the cross-validation procedure (which is considered good practice) or on the same test data used for cross-validation (Figure 1). As previously mentioned, because the above cross-validation procedure takes the average of the validation metric across trials, the models are not biased toward the test data used for cross-validation. Thus, it is legitimate to report model performance based on these data because testing on new unseen data will likely yield the same result.

While the *mTRFpredict* function outputs the same performance metrics as *mTRFcrossval*, it also outputs the predicted signal for further evaluation. When predicting a multivariate signal such as EEG, a performance measure is calculated for every feature (i.e., EEG channel), allowing the user to base evaluation of the model on whichever features they deem most relevant.

## Examples

The examples presented in this section use data from a published study that measured EEG responses of human subjects to natural, continuous speech (Di Liberto et al., 2015). The subject listened to an audiobook version of a classic work of fiction read by a male speaker in American English. The audio was presented in 28 segments (each ~155 s in duration), of which a subset of five are used in the examples in this chapter. EEG data were recorded using a 128-channel ActiveTwo system (BioSemi) and digitized at a rate of 512 Hz. Offline, the data were digitally filtered between 1 and 15 Hz, downsampled to a rate of 128 Hz and re-referenced to the left and right mastoid channels. Only 32 of the 128 channels recorded are included in the analysis, but crucially, are distributed evenly across the head (Mirkovic et al., 2015). Further details can be found in the original study (Di Liberto et al., 2015).

This section details several examples that demonstrate how the mTRF Toolbox can be used to relate neural data to sensory stimuli in a variety of different ways. These include:

1. Univariate TRF estimation
2. Optimization and prediction
3. Multivariate TRF analysis
4. Stimulus reconstruction
5. Multimodal TRF estimation
6. TRF vs. cross-correlation

While the examples all relate to EEG data collected during speech stimuli, as stated earlier, these approaches can all be used with other types of sensory stimuli.

### Univariate TRF estimation

**Figure 2:**
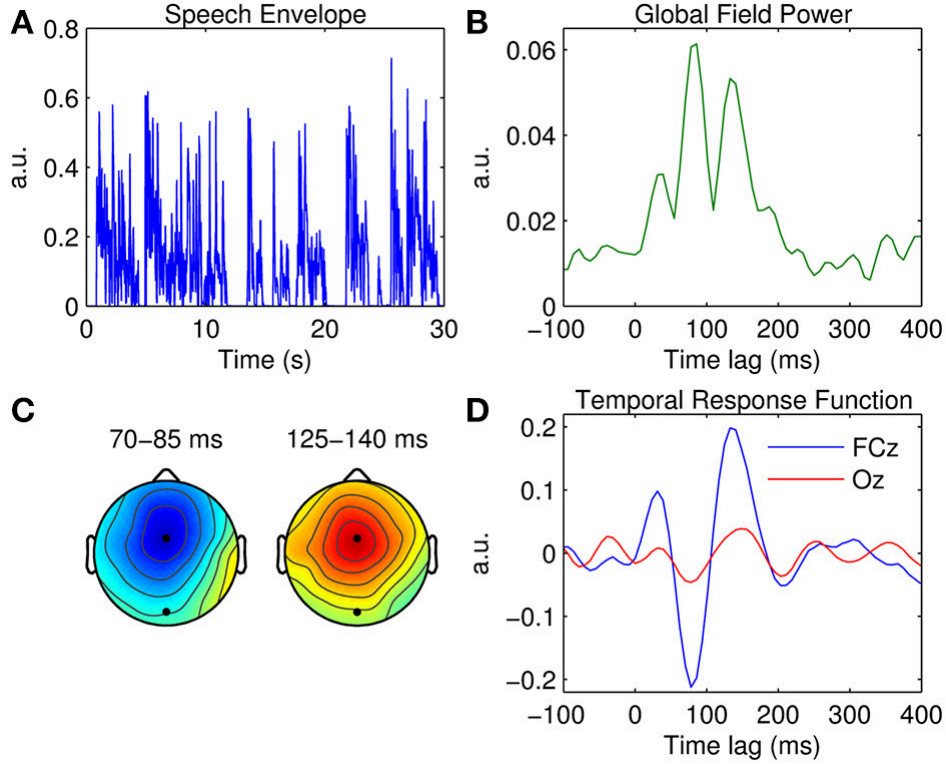
**Univariate TRF estimation. (A)** A 30-s segment of the broadband speech envelope. **(B)** Global field power measured at each time lag. **(C)** Scalp topographies of the dominant TRF components occurring at ~80 and ~140 ms. The black markers indicate the locations of fronto-central channel, FCz, and occipital channel, Oz. **(D)** Grand average TRFs at FCz (blue trace) and Oz (red trace).

The aim here is to estimate the temporal response function that maps a univariate representation of the speech envelope onto the EEG signal recorded at each channel. The broadband envelope of the speech signal (Figure 2A) was calculated using:

(11) $$x_{a}(t)=x(t)+j\hat{x}(t),$$

where *x*_*a*_(*t*) is the complex analytic signal obtained by the sum of the original speech *x*(*t*) and its Hilbert transform $\hat{x}\left(t\right)$. The envelope was defined as the absolute value of *x*_*a*_(*t*). This was then downsampled to the same sampling rate as the EEG data, after applying a zero-phase shift anti-aliasing filter. TRFs were calculated between lags of −150 and 450 ms, allowing an additional 50 ms at either end for regression artifacts. An estimate was computed separately for each of the five trials and then averaged. The ridge parameter was empirically chosen to maintain component amplitude (Lalor et al., 2006).

A measure of global field power (GFP) was first estimated by calculating TRF variance across the 32 channels (Figure 2B). GFP constitutes a reference-independent measure of response strength across the entire scalp at each time lag (Lehmann and Skrandies, 1980; Murray et al., 2008). Based on the temporal profile of the GFP measure, three clear TRF components are evident at ~50, ~80, and ~140 ms. Figure 2C shows the scalp topographies of the latter two of these components. Their latency and polarity resemble that of the classic N1 and P2 components of a typical (mastoid-referenced) auditory-evoked response. The topography of the N1-P2 complex suggests that both components are strongest at fronto-central position FCz. The grand average TRF calculated at FCz is shown in Figure 2D, along with the TRF measured at occipital location Oz for comparison.

### Optimization and prediction

The aim here is to use the TRF model to predict the EEG response of unseen data. This time, tuning of model parameters was conducted using a more systematic approach, i.e., that of the cross-validation procedure described earlier. Specifically, TRFs were calculated for a range of ridge values (λ = 2^0^, 2^2^, …, 2^20^) on each of the separate trials. For each ridge value, the TRFs were averaged across every combination of four trials and used to predict the EEG of the remaining fifth trial. Here, the data were modeled at time lags between 0 and 200 ms as these lags reflected the most information in the global TRF responses (Figure 2B). Inclusion of additional lags (pre-stimulus or post-stimulus) did not improve model performance.

**Figure 3:**
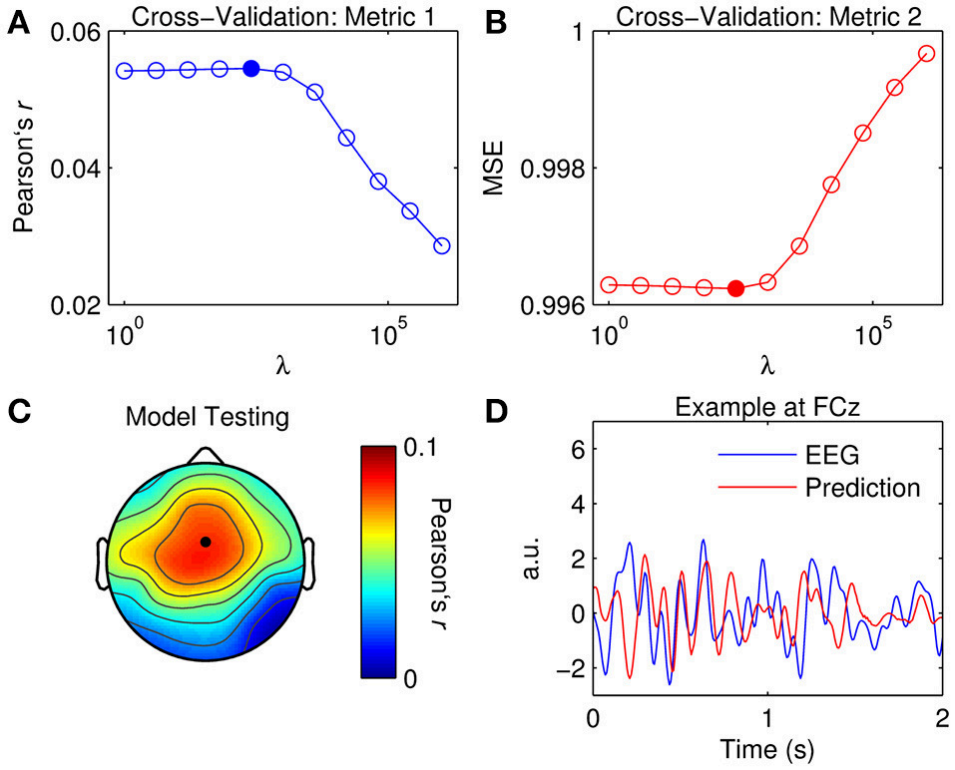
**Optimization of TRFs for EEG prediction. (A)** Cross-validation of model based on the correlation between the original and predicted EEG response (Pearson's r averaged across channels and trials). The filled marker indicates the highest *r*-value, i.e., the optimal ridge value. **(B)** Cross-validation based on mean squared error (MSE). The optimal ridge value is identified by the lowest MSE-score. **(C)** Test of the optimized TRF model shows the correlation coefficient at each channel. The black marker indicates the location of channel FCz. **(D)** Two-second segments of the EEG response at FCz (blue trace) and the corresponding estimate predicted by the optimized TRF model (red trace).

Figure 3A shows the results of the cross-validation based on the correlation coefficient (Pearson's *r*) between the original and predicted EEG responses. Critically, the *r*-values were averaged across the five trials to prevent overfitting the model to the test data. The *r*-values were also averaged across the 32 channels such that model performance would be optimized in a more global manner. Alternatively, one could average across only channels within a specified top percentile or based on a specific location. Figure 3B shows the results of the cross-validation based on the mean squared error. The same averaging procedure was used to identify the optimal ridge value here.

The ridge value was chosen such that it maximized the correlation between the original and predicted EEG (David and Gallant, 2005). Note that using MSE as a criteria for cross-validation would have yielded the same result. Figure 3C shows the correlation coefficient obtained at each channel using the optimized TRF model. The topographical distribution of Pearson's *r* is very similar to that of the dominant TRF components (Figure 2C). Indeed, it is unsurprising that the model performed best at channels where the response was strongest. Figure 3D shows 2-s segments of the EEG response at FCz and the corresponding estimate predicted by the optimized TRF model.

### Multivariate TRF analysis

**Figure 4:**
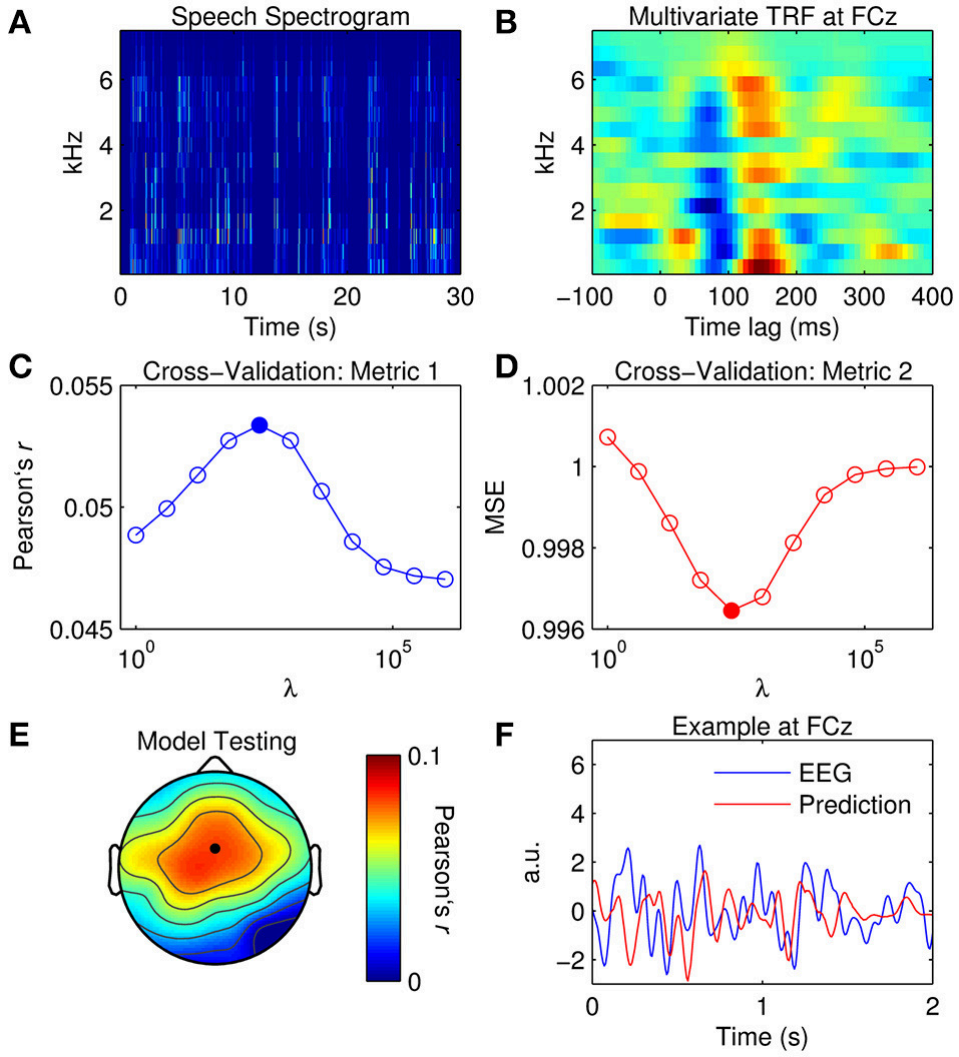
**Multivariate TRF estimation and EEG prediction. (A)** A 30-s segment of the speech spectrogram. **(B)** Grand average mTRF at channel FCz. **(C)** Cross-validation of model based on the correlation between the original and predicted EEG response (Pearson's *r* averaged across channels and trials). The filled marker indicates the highest *r*-value, i.e., the optimal ridge value. **(D)** Cross-validation based on mean squared error (MSE). The optimal ridge value is identified by the lowest MSE-score. **(E)** Test of the optimized mTRF model shows the correlation coefficient at each channel. The black marker indicates the location of channel FCz. **(F)** Two-second segments of the EEG response at FCz (blue trace) and the corresponding estimate predicted by the optimized TRF model (red trace).

The aim here is to estimate the TRF for a multivariate (spectrogram) representation of speech, i.e., an mTRF. The spectrogram representation (Figure 4A) was obtained by first filtering the speech stimulus into 16 logarithmically-spaced frequency bands between 250 and 8 kHz according to Greenwoods equation (Greenwood, 1990). Filtering the data in a logarithmic manner attempts to model the frequency analysis performed by the auditory periphery. The energy in each frequency band was calculated using a Hilbert transform as above (Equation 11).

For visualization, mTRFs were calculated between lags of −150 and 450 ms and model parameters were tuned empirically. Figure 4B shows the mTRF response at channel FCz for all frequency bands between 250 and 8000 Hz. Visual inspection of Figure 4B suggests that the dominant N1_TRF_ and P2_TRF_ components encoded speech information at nearly every frequency band up to ~6 kHz, which is where most of the information was contained in the speech signal (Figure 4A). Averaging the mTRF across frequency bands would yield a univariate TRF measure that closely approximates the TRF calculated using the broadband envelope (Figure 2D).

To predict the EEG response with the mTRF model, the same approach was implemented as before. Although the results yielded by the cross-validation (Figures 4C,D) were similar to those for the univariate TRF approach (Figures 3A,B), the mTRF approach appeared to be more sensitive to changes in the ridge value. Further investigation revealed that this could not be attributed to using different regularization penalties in univariate and multivariate analyses. Despite this, performance of the optimized mTRF model was akin to that of the univariate TRF model over the entire scalp (Figures 4E,F).

While it has been demonstrated that multivariate TRF models are superior to univariate TRF models for predicting EEG responses (Di Liberto et al., 2015), it must be taken into consideration that multivariate TRF analysis is more sensitive to regularization (certainly for ridge regression) and can involve considerably more computations.

### Stimulus reconstruction

The aim here is to generate a decoder that models the data in the backwards direction (i.e., from EEG to stimulus) and to use it to reconstruct an estimate of the univariate stimulus input. The advantages of this approach over the forward modeling technique are outlined in the Introduction and Backward Models Section. Tuning of model parameters was conducted using the same cross-validation technique described for the TRF models. Specifically, decoders were calculated for the same range of ridge values (λ = 2^0^, 2^2^, …, 2^20^) at time lags between 0 and 200 ms. The difference here was that the EEG was treated as the “input” and the stimulus as the “output,” and the direction of the lags was reversed, i.e., −200 to 0.

**Figure 5:**
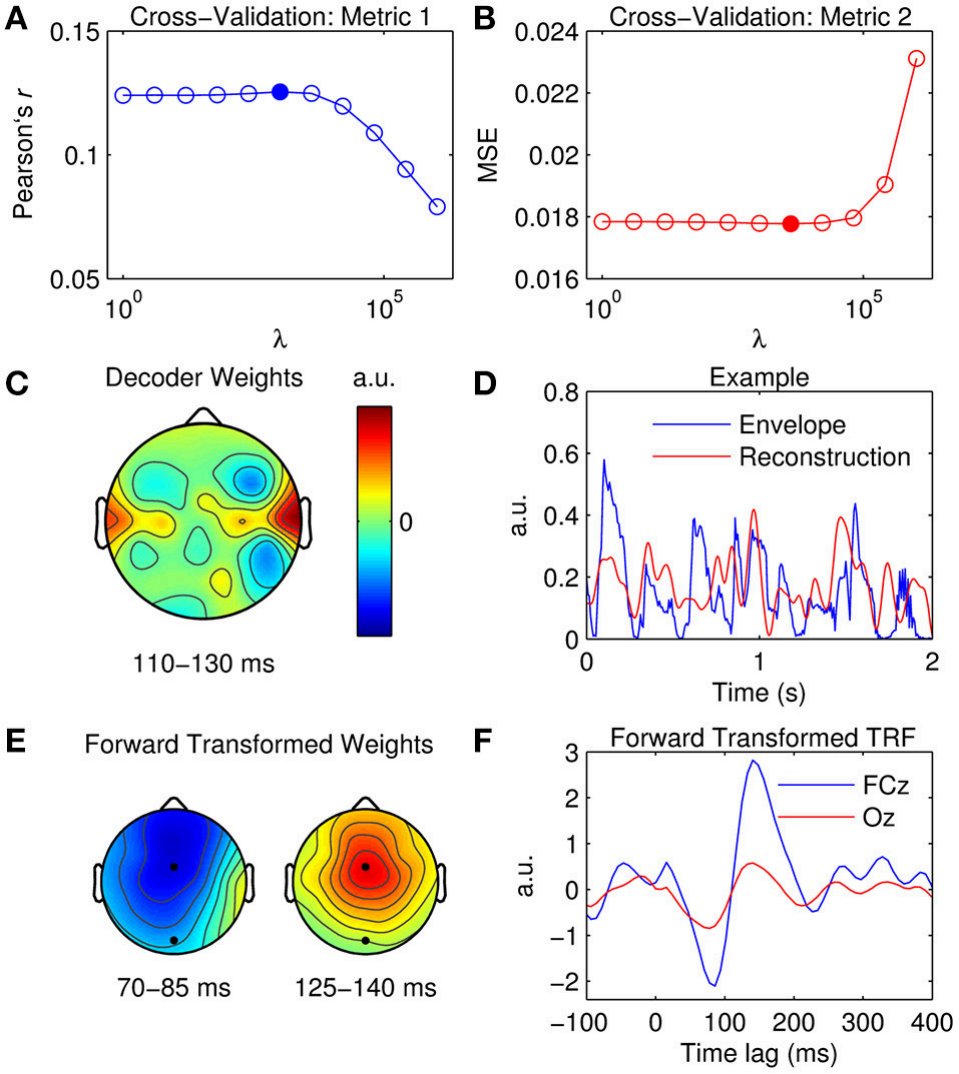
**Stimulus reconstruction. (A)** Cross-validation of model based on the correlation between the original and reconstructed speech envelope (Pearson's *r* averaged across trials). The filled marker indicates the highest *r*-value, i.e., the optimal ridge value. **(B)** Cross-validation based on mean squared error (MSE). The optimal ridge value is identified by the lowest MSE-score. **(C)** Decoder channel weights averaged over time lags between 110 and 130 ms. **(D)** Two-second segments of the original speech envelope (blue trace) and the corresponding estimate reconstructed by the optimized decoder (red trace). **(E)** Decoder channel weights transformed to forward model space using the inversion procedure described by Haufe et al. (2014). The black markers indicate the locations of fronto-central channel, FCz, and occipital channel, Oz. **(F)** Temporal response function obtained by inverting the decoder weights to the forward model domain at FCz (blue trace) and Oz (red trace).

Figure 5A shows the results of the cross-validation as measured by the correlation coefficient between the original and reconstructed speech envelope, while Figure 5B represents validation of the model ridge parameter based on MSE. Again, both metrics have been averaged across trials to prevent overfitting to the test data. All 32 EEG channels were included in the model validation procedure to optimize performance. The advantages of the backward modeling approach over forward modeling are evidenced by the dramatic reduction in residual error as indexed by the correlation values. This is mainly attributable to the fact that the decoder can utilize information across the entire head simultaneously (i.e., in a multivariate sense) to determine the speech estimate, whereas when modeling in the forward direction, the predicted EEG estimate is based on a single univariate mapping between the stimulus and the EEG response at that specific channel (Mesgarani et al., 2009). Additionally, the predictions for the forward modeling approach are evaluated in the EEG domain, where the low SNR negatively affects prediction accuracy. In contrast, the backward modeling approach moves the estimation of these measures to the stimulus domain, which is defined by the experimenter. Therefore, in case of stimuli with low SNR (e.g., a speech envelope), the quality of fit will likely be higher for backward modeling.

While the decoder channel weights are not readily interpretable in a neurophysiological sense, their weighting reflects the channels that contribute most toward reconstructing the stimulus signal (Haufe et al., 2014). Figure 5C shows the decoder weights averaged across time lags between 110 and 130 ms (this was where weighting was maximal as indexed by GFP). In comparison to the TRF topographies (Figure 2C), the distribution of model weight is much more localized. Because the decoder is not required to encode information at every channel across the scalp as a TRF does, it can selectively weight only those channels important for reconstruction, whilst ignoring irrelevant and noisy channels by giving them a lower weighting (Haufe et al., 2014). A 2-s sample of a reconstructed estimate can be seen in Figure 5D. Stimulus reconstruction for a multivariate stimulus is conducted in much the same manner, except model performance must be evaluated for every feature (e.g., frequency band) separately or by averaging across features and then evaluating.

Previous research has described a procedure that enables neurophysiological interpretation of backward model coefficients (Haufe et al., 2014). Specifically, they proposed a deterministic approach to transform previously fit linear backward model coefficients into linear forward model coefficients. This procedure enables the neurophysiological interpretation of the parameters of linear backward models which could be otherwise misleading and erroneous. The *mTRFtransform* function implements this procedure specifically for backward models derived using the ridge regression technique (e.g., Figures 5E,F).

### Multimodal TRF estimation

**Figure 6:**
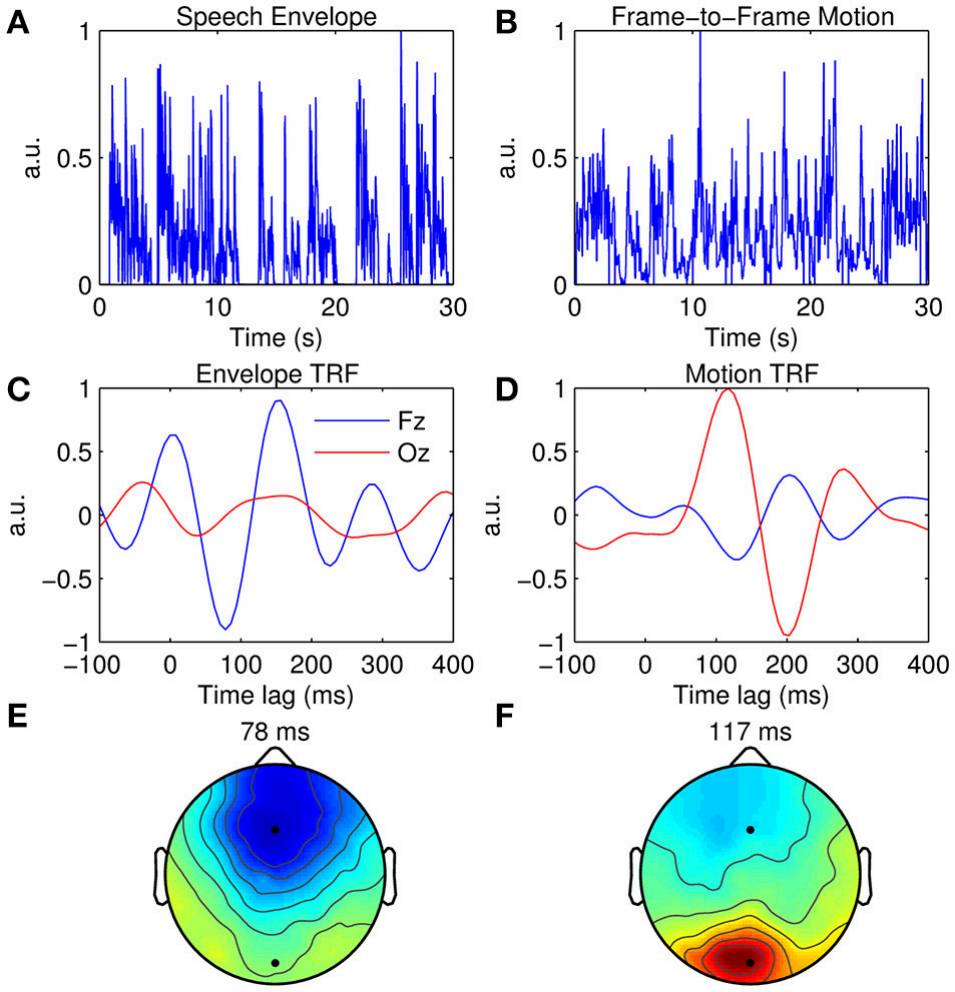
**Multimodal TRF estimation. (A)** A 30-s segment of the broadband speech envelope. **(B)** A 30-s segment of the corresponding frame-to-frame visual motion. **(C)**, Grand average envelope TRFs at Fz (blue trace) and Oz (red trace). **(D)**, Grand average motion TRFs at Fz (blue trace) and Oz (red trace). **(E)** Scalp topography of the dominant envelope TRF component occurring at 78 ms. **(F)** Scalp topography of the dominant motion TRF component occurring at 117 ms.

As well as extracting the neural response to unimodal stimuli, the TRF approach can be used to disentangle contributions from multimodal signals (or multiple signals within the same modality such as a cocktail party scenario, e.g., Power et al., 2012). This can be demonstrated using EEG recorded during natural audiovisual speech. The data presented here were published in a study that investigated the influence of visual speech on the cortical representation of auditory speech (Crosse et al., 2015a). The subject listened to 15 min of natural audiovisual speech, of which a subset of 7 min are used here. The auditory stimulus was characterized as the broadband envelope as before (Figure 6A), while the visual stimulus was characterized by calculating the frame-to-frame motion of the videos (Figure 6B). For each frame, a matrix of motion vectors was calculated using an “Adaptive Rood Pattern Search” block matching algorithm (Barjatya, 2004). A measure of global motion flow was obtained by calculating the sum of all motion vector lengths of each frame (Bartels et al., 2008). This was then converted from an RGB representation to relative luminance and upsampled from 30 to 128 Hz to match the rate of the EEG data.

Figure 6C shows the TRFs at channels Fz and Oz when the acoustic envelope is mapped to the EEG, whereas Figure 6D shows the TRFs at the same channels when the visual motion signal is mapped to the same EEG data. TRF amplitude is much greater at Fz than at Oz when the auditory signal is used, whereas the converse is true for the visual signal. This can also be seen in the topographies which show a dominant response over frontal scalp for the envelope TRF (Figure 6E) and a dominant response over occipital scalp for the motion TRF (Figure 6F). Although the same EEG data were analyzed in both cases, responses from different sensory cortical regions could be extracted by simply mapping from features specific to each sensory modality.

To measure multisensory integration, the *mTRFmulticrossval* function can be used to fit an “additive model” based on the algebraic sum of the unisensory model coefficients (Stein and Meredith, 1993). The additive model is tested on the multisensory neural response data and its performance can then be compared with that of the multisensory model to obtain an objective measure of integration. For further detail, see Crosse et al. (2015a).

### TRF vs. cross-correlation

As mentioned earlier, the impulse response of an LTI system can be easily approximated via a simple cross-correlation of the input and output. While this approach is more straightforward than using techniques such as normalized reverse correlation or ridge regression, it is only suitable for input signals that conform to a stochastic process. To demonstrate this empirically, a comparison is made between each of these approaches using both speech and white noise as a stimulus input signal. The speech data presented here are the same as those in the previous examples. The non-speech data presented here were published in a study that investigated the TRF approach for estimating the response of the auditory system to Gaussian white noise (Lalor et al., 2009). The subject listened to ten 120-s segments of uninterrupted noise stimuli, of which a subset of six are used in this example. The stimuli were Gaussian broadband noise with energy limited to a bandwidth of 0–22.05 kHz, modulated using Gaussian noise signals with uniform power in the range 0–30 Hz. To account for the logarithmic nature of auditory stimulus intensity perception, the values of these modulating signals, *x*, were then mapped to the amplitude of the audio stimulus, *x*′, using the following exponential relationship:

(12) $$x\prime =10^{2x}.$$

EEG data were recorded and processed using the exact same procedure described in the previous examples. Further details can be found in the original study (Lalor et al., 2009).

**Figure 7:**
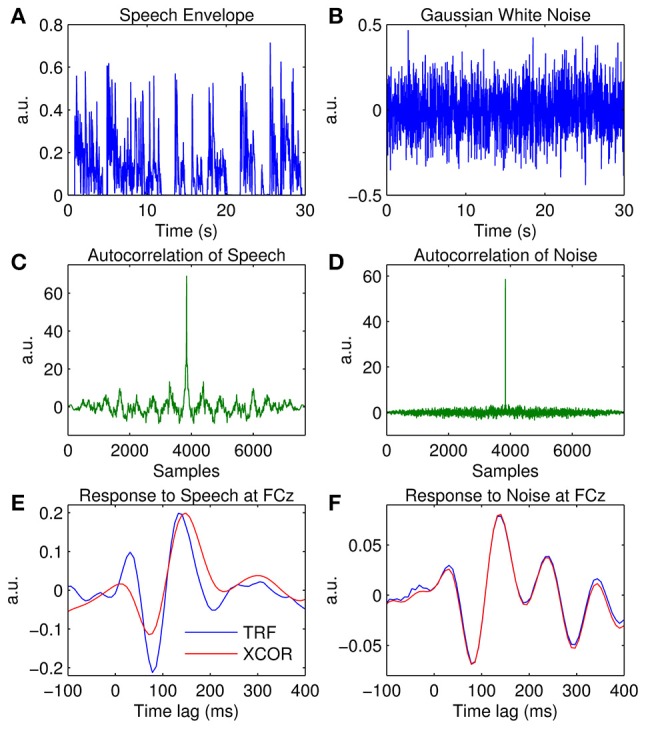
**Comparison of the temporal response function (TRF) and cross-correlation (XCOR) approach. (A)** A 30-s segment of the broadband speech envelope. **(B)** A 30-s segment of amplitude modulated noise. **(C)**, Autocorrelation of the speech envelope. **(D)**, Autocorrelation of the noise signal. **(E)** The impulse response to speech at channel FCz estimated using the TRF approach (blue trace) and the cross-correlation approach (red trace). **(F)** The impulse response to white noise at channel FCz estimated using the TRF approach (blue trace) and the cross-correlation approach (red trace).

Examples of the speech and noise stimuli used in the experiments are shown in Figures 7A,B respectively. The autocorrelation of each stimulus reveals that the speech stimulus is correlated with itself at multiple time lags (Figure 7C), whereas the noise stimulus is only correlated with itself at a zero time lag (Figure 7D). Figure 7F shows the impulse response for the white noise stimulus calculated at channel FCz using the TRF approach and the cross-correlation (XCOR) approach. Visual inspection suggests that the cross-correlation and TRF approaches produce approximately identical estimates of the system response function. However, the same was not true for the speech stimulus, where the cross-correlation approach caused temporal smearing of the impulse response estimate compared to the TRF approach (Figure 7E). This is because the stimulus dynamics map to the EEG signal at multiple overlapping time lags. This demonstrates the utility of the TRF technique for characterization of sensory systems in response to slowly-modulating naturalistic stimuli such as human speech.

## Discussion

Here, we have described a new MATLAB-based SI toolbox for modeling the relationship between neural signals and natural, continuous stimuli. The above examples demonstrate how this versatile toolbox can be applied to both univariate and multivariate datasets, as well as unisensory and multisensory datasets. Importantly, it can also be used to map in both the forwards and backwards direction to perform response function estimation and stimulus reconstruction respectively, providing complementary analysis techniques.

### Applications

The mTRF Toolbox has many applications in sensory neuroscience, none more so than for studying how natural speech is processed in the human brain. The forward TRF approach has previously been used to demonstrate how neural responses to uninterrupted speech can be extracted with precise temporal resolution in humans using both intracranial and non-invasive recording techniques (Lalor and Foxe, 2010). Subsequent studies using this approach have yielded several key findings relating to how the brain selectively attends to a single speech stream in a cocktail party scenario (Power et al., 2012) and how spectrotemporal and phonetic information are represented in auditory cortical activity (Di Liberto et al., 2015). Other applications of the toolbox include using both backward and forward models to investigate audiovisual speech processing (Crosse et al., 2015a, 2016) and visual speech processing, i.e., speech reading (Crosse et al., 2015b). Alternative SI techniques (that ultimately yield the same solution) have also been used to investigate auditory scene analysis (Ding and Simon, 2012a; Mesgarani and Chang, 2012; Zion-Golumbic et al., 2013; O'Sullivan et al., 2015), speech-in-noise (Ding and Simon, 2013; Ding et al., 2014), overt and covert cortical representations of speech (Martin et al., 2014) and detailed spectrogram reconstructions of speech from intracranial recordings (Pasley et al., 2012).

Aside from studying speech, the forward TRF approach has been applied in vision research to study how the human brain processes stimuli that modulate in contrast over time (Lalor et al., 2006, 2007; Frey et al., 2010; Murphy et al., 2012). This particular approach has also been used in clinical research to investigate visual processing deficits in children with autism spectrum disorder (Frey et al., 2013) and in adults with schizophrenia (Lalor et al., 2008, 2012). More recently, it has been modified to studying how the brain processes more naturalistic visual stimuli such as coherent motion (Gonçalves et al., 2014). In addition to characterizing mappings between visual stimulus features and EEG recordings, researchers have recently reconstructed finger movements from surface EMG signals using the same regularized linear regression approach (Krasoulis et al., 2015), further demonstrating the versatility of this technique.

### Considerations

The linear assumption underlying the reverse correlation method has implications for its interpretation. This assumption of a linear relationship between stimulus feature and neural response amplitude likely results in a response measure reflective of feedforward activity in a subset of cortical cells (Lalor et al., 2009). Thus, it is possible that such an approach is insensitive to cortical responses that relate to the stimulus in a non-linear manner including lateral and feedback contributions, which may have implications for studying the effects of higher-order cognitive processes. This is in contrast to the challenge involved in disambiguating the myriad feedforward, lateral and feedback contributions to the time-locked average ERP (Di Russo et al., 2005).

Indeed, such linear assumptions will need to be addressed in order to accurately characterize populations of neurons that respond in a non-linear way to complex stimuli (Theunissen et al., 2000). That said, a previous study that implemented a quadratic extension of the linear TRF approach for modeling visual responses to contrast stimuli did not find any significant improvement in model performance relative to that of a linear model (Lalor et al., 2008). Subsequent studies that applied the same quadratic model to the auditory system did however demonstrate marginal improvements in model performance for acoustic white noise stimuli (Power et al., 2011a,b). Expansion of the TRF model into higher orders has also been explored using machine learning techniques such as support vector regression, but similarly, yielded only negligible improvements (Crosse, 2011). While such non-linear regression techniques can result in slight improvements in model performance, there is a considerable trade-off between performance and computation time that often make them impractical.

However, the fact that non-linear models perform only marginally better than linear models for population data (e.g., EEG; Power et al., 2011a,b), and yet appear to be more beneficial for modeling single-unit data (e.g., ECoG; Theunissen et al., 2000) may imply something fundamental about the nature of EEG recordings. Each EEG electrode detects neural activity from large cortical populations (10^7^–10^9^ neurons) due to the spatial smearing effects of volume conduction (Freeman et al., 2003). Thus, activation patterns that are common across the largest neural populations will contribute most to the signal recorded at the scalp. Because of the diversity of non-linear responses across neurons, it is likely that such activity is encoded in small, sub-populations of neurons, whereas linear responses are likely encoded on a more macroscopic level. The effects of volume conduction could therefore result in much of this non-linear activity being obscured in the resulting EEG recording. Indeed if this were the case, it would explain why linear regression techniques perform comparably to that of non-linear techniques for modeling EEG responses. In support of this notion, other EEG/MEG modeling algorithms such as SPoC (Dähne et al., 2014)—which relates the amplitude of neural oscillations to stimulus features or behaviorally relevant parameters—have specifically used linear models based on the fact that superposition of such oscillations is known to be linear and instantaneous (Parra et al., 2005; Nunez and Srinivasan, 2006).

## Ethics statement

The Ethics Committee of the Health Sciences Faculty at Trinity College Dublin. Written informed consent was obtained during testing.

## Author contributions

The toolbox and paper were conceived by MC, GD, and EL. MC, GD, AB, and EL designed and wrote the toolbox code. MC, GD collected and analyzed the sample data. MC, GD, AB, and EL wrote the manuscript.

### Conflict of interest statement

The authors declare that the research was conducted in the absence of any commercial or financial relationships that could be construed as a potential conflict of interest.
